# Subclinical tuberculosis among adults with HIV: clinical features and outcomes in a South African cohort

**DOI:** 10.1186/s12879-018-3614-7

**Published:** 2019-01-05

**Authors:** Kristina L. Bajema, Ingrid V. Bassett, Sharon M. Coleman, Douglas Ross, Kenneth A. Freedberg, Anna Wald, Paul K. Drain

**Affiliations:** 1 Department of Medicine, University of Washington, 1959 NE Pacific St., Box 356429, Seattle, WA 98195 USA; 2 Department of Medicine, Massachusetts General Hospital, Harvard Medical School, Boston University School of Public Health, Boston, USA; 3 Boston University School of Public Health, Boston, USA; 4 Department of Medicine, St. Mary’s Hospital, Durban, South Africa; 5 Departments of Medicine, Epidemiology, and Laboratory Medicine, Vaccine and Infectious Disease Division, Fred Hutchinson Cancer Research Center, University of Washington, Seattle, USA; 6 Departments of Medicine, Global Health, and Epidemiology, University of Washington, Seattle, USA; 7 Departments of Surgery and Medicine, Massachusetts General Hospital, Harvard Medical School, Boston, USA

**Keywords:** Tuberculosis, Subclinical infections, HIV, AIDS-related opportunistic infections, Disease progression

## Abstract

**Background:**

Subclinical tuberculosis is an asymptomatic disease phase with important relevance to persons living with HIV. We describe the prevalence, clinical characteristics, and risk of mortality for HIV-infected adults with subclinical tuberculosis.

**Methods:**

Untreated adults with HIV presenting for outpatient care in Durban, South Africa were screened for tuberculosis-related symptoms and had sputum tested by acid-fast bacilli smear and tuberculosis culture. Active tuberculosis and subclinical tuberculosis were defined as having any tuberculosis symptom or no tuberculosis symptoms with culture-positive sputum. We evaluated the association between tuberculosis disease category and 12-month survival using Cox regression, adjusting for age, sex, and CD4 count.

**Results:**

Among 654 participants, 96 were diagnosed with active tuberculosis disease and 28 with subclinical disease. The median CD4 count was 68 (interquartile range 39–161) cells/mm^3^ in patients with active tuberculosis, 136 (72–312) cells/mm^3^ in patients with subclinical disease, and 249 (125–394) cells/mm^3^ in those without tuberculosis disease (*P < 0.001*). The proportion of smear positive cases did not differ significantly between the subclinical (29%) and active tuberculosis groups (14%, *P 0.08*). Risk of mortality was not increased in individuals with subclinical tuberculosis relative to no tuberculosis (adjusted hazard ratio 0.84, 95% confidence interval 0.26–2.73).

**Conclusions:**

Nearly one-quarter of tuberculosis cases among HIV-infected adults were subclinical, which was characterized by an intermediate degree of immunosuppression. Although there was no significant difference in survival, anti-tuberculous treatment of subclinical cases was common.

**Trial registration:**

Prospectively registered on ClinicalTrials.gov, NCT01188941 (August 26, 2010).

## Background

In 2016, over 10 million people worldwide developed active tuberculosis (TB) [1]. TB was the leading infectious disease cause of mortality as well as the leading cause of HIV-related mortality. Early diagnosis of highly contagious individuals as well as those at risk for progressing from latent infection to active disease may be critical for controlling the TB epidemic.

The traditional dichotomy of latent versus active TB has been more accurately modeled as a spectrum of infection whereby weakened host innate and acquired immune responses, as occur in HIV, allow mycobacterial replication [2, 3]. In the setting of increasing bacillary burden and host damage, clinical symptoms may develop. The preceding asymptomatic period during which viable *M. tuberculosis* can be detected in the host using existing microbiologic or radiologic tests has been described as subclinical TB. This disease state occurs on a continuum between latent and active disease [4–6].

The World Health Organization currently recommends all persons living with HIV (PLHIV) be screened for TB disease at each clinical encounter by assessing four TB-related symptoms: current cough, fever, night sweats, or weight loss [7]. Those who screen negative are eligible for isoniazid preventive therapy, while those who screen positive require further investigation for active TB disease. The overall sensitivity of this screen among PLHIV is estimated at 79% (95% confidence interval [CI] 58–91%) [8]. Persons with subclinical TB will be missed by this approach, potentially placing others at risk for infection and allowing disease to progress to active TB. In the HIV-coinfected population, progression may occur rapidly, thus raising the concern for higher risk of mortality among those with subclinical TB compared to latent TB [8–10]. It is not known how survival outcomes for subclinical disease compare with active disease. Given these potential implications, it is important to better characterize this asymptomatic disease state. We describe the prevalence, clinical and laboratory findings, and risk of death in a cohort of untreated (antiretroviral therapy [ART]-naïve) HIV-infected adults with subclinical TB in South Africa.

## Methods

### Study design

We enrolled untreated adults with HIV between October 2011 and January 2014 at four outpatient sites in KwaZulu-Natal, South Africa as previously described [11, 12]. Non-pregnant adults ≥18 years who had not received anti-tuberculous therapy (ATT) within three months were eligible for enrollment. No minors were included. The study was approved by the McCord Hospital Medical Research Ethics Committee, St. Mary’s Hospital Medical Research Ethics Committee, the University of KwaZulu-Natal Biomedical Research Ethics Committee, Partners Institutional Review Board, and the University of Washington Human Subjects Division. All participants provided written informed consent.

### Procedures

At enrollment, study nurses recorded demographic and clinical information including TB-related symptoms: cough, fever, night sweats, and weight loss. Participants underwent CD4 T-cell count testing and provided a single expectorated sputum specimen; those unable to provide a sample underwent sputum induction with 3% hypertonic saline delivered by nebulizer. Specimens were transported daily to the TB laboratory at the University of KwaZulu-Natal for concentrated AFB smear and mycobacterial culture. Acid-fast bacilli (AFB) smear was performed using both Ziehl-Neelsen and auramine staining with fluorescence microscopy and graded according to standard criteria [13]. Culture was performed using both solid Middlebrook 7H11 agar and Bactec mycobacterial growth indicator tubes (MGIT) 960 system. Cases were considered culture positive based on identification of *M. tuberculosis* in either solid or liquid media. Participant urine samples were also tested for lipoarabinomannan (LAM) using the Determine™ TB LAM assay (Alere Inc.) [11]. LAM positive cases were designated as grade 1 or higher on using the manufacturer’s 5-grade reference card. All participants were offered treatment for HIV and TB according to South African Department of Health and WHO guidelines (ART initiated at CD4 ≤ 350 cells/mm^3^ for groups with TB disease and CD4 ≤ 200 cells/mm^3^ for groups without TB) [14, 15].

Outcomes were assessed 12 months after enrollment through review of clinical site TB registers and the Department of Health’s TB Control Programme. Information was recorded on TB treatment initiation, completion, default, failure, and death according to the South African Population Register [16].

### Statistical analysis

Participants were categorized as having active TB disease, subclinical TB disease, or no microbiologic TB disease. Active TB cases were defined as having a positive *M. tuberculosis* culture in addition to the presence of at least one TB-related symptom. Subclinical TB cases were defined as having a positive *M. tuberculosis* culture but no TB-related symptoms. No microbiologic TB disease was defined as having no microbiologic evidence of *M. tuberculosis* by smear or culture and further subcategorized into those who were and were not treated empirically with multi-drug ATT at the discretion of the clinician, denoted as “no microbiologic TB/ATT” and “no microbiologic TB/no ATT” respectively.

Statistical methods used included Fisher’s exact test, one-way ANOVA, and Kruskal-Wallis test to compare baseline demographic, clinical, and laboratory characteristics between participants with active or subclinical TB and no microbiologic TB disease. Kaplan-Meier curves were used to display survival across TB diagnosis groups. We also evaluated the association between TB category and 12-month survival outcomes using Cox regression and adjusting for age, sex, and categorical CD4 count. We calculated both unadjusted and adjusted hazard rates and 95% CIs. In further analysis, we evaluated mortality outcomes in subgroups delineated by urine LAM status and ATT initiation. We also evaluated mortality according to baseline ART eligibility. The proportional hazards assumption was tested using log-log plots and time-varying predictors. Analyses were conducted in R [17].

## Results

We enrolled 727 untreated PLHIV. After excluding 73 persons for whom mycobacterial culture and AFB smear data were missing, we included 654 participants for analysis. Mean age was 34 years, 348 (53%) were men, 160 (25%) currently smoked tobacco, and 53 (8%) reported prior treatment for TB (Table 1). At baseline, 243 (37%) had no TB-related symptoms, 139 (21%) had one symptom, 116 (18%) had two symptoms, 85 (13%) had three symptoms, and 71 (11%) had four symptoms. Median CD4 cell count was 206 (interquartile range [IQR] 81–349) cells/mm^3^. AFB smear was positive in 21 individuals (3%), mycobacterial culture was positive in 124 (19%), and urine LAM was positive in 99 (15%).

**Table 1:** Characteristics of adults with HIV stratified by tuberculosis status

	All Individuals *N=654*	Active TB *N=96*	Subclinical TB *N=28*	No Microbiologic TB/ATT^1^ *N=40*	No Microbiologic TB/No ATT *N=490*	*P* Value
Demographics						
Mean age, years (SD)	34 (9)	35 (9)	33 (9)	36 (11)	34 (10)	*0.55* ^*2*^
Men	348 (53%)	65 (68%)	14 (50%)	21 (53%)	248 (51%)	*0.02*
Education: high school or higher	249 (38%)	34 (36%)	8 (29%)	12 (30%)	195 (40%)	*0.39*
Marital status						
Never married	542 (83%)	83 (87%)	22 (79%)	32 (80%)	405 (83%)	*0.53*
Currently married	89 (14%)	9 (9%)	6 (21%)	7 (18%)	67 (14%)	*0.29*
Clinical						
Current tobacco	160 (25%)	19 (20%)	7 (25%)	8 (20%)	126 (26%)	*0.61*
Prior TB treatment	53 (8%)	8 (8%)	0	3 (8%)	42 (9%)	*0.34*
TB-related symptoms						
None	243 (37%)	0	28 (100%)	7 (18%)	208 (42%)	--
Any 1 symptom	139 (21%)	23 (24%)	0	11 (28%)	105 (21%)	--
Any 2 symptoms	116 (18%)	73 (76%)	0	22 (55%)	177 (36%)	--
Any 3 symptoms	85 (13%)	53 (55%)	0	14 (35%)	89 (18%)	--
All 4 symptoms	71 (11%)	23 (24%)	0	6 (15%)	42 (9%)	--
Median CD4 cell count (IQR), cells/mm^3^	206 (81 – 349)	68 (39 – 161)	136 (72 - 312)	90 (40 – 180)	249 (125 – 394)	*<0.001*
<100	170 (30%)	55 (62%)	10 (37%)	21 (54%)	84 (20%)	*<0.001*
100-200	108 (19%)	18 (20%)	8 (30%)	9 (23%)	73 (18%)	*0.36*
>200	289 (51%)	16 (18%)	9 (33%)	9 (23%)	255 (62%)	*<0.001*
Eligible for ART initiation^3^	288 (51%)	79 (89%)	22 (81%)	30 (77%)	157 (38%)	*<0.001*
TB test results						
AFB smear positive	21 (3%)	13 (14%)	8 (29%)	0	0	*0.08*
Low	2	0	2			
+	9	6	3			
++	3	2	1			
+++	7	5	2			
*M. tuberculosis* culture	124 (19%)	96 (100%)	28 (100%)	0	0	*--*
positive						
Urine LAM positive	99 (15%)	35 (37%)	7 (25%)	9 (23%)	48 (10%)	*<0.001*

Abbreviations: *AFB* acid fast bacilli, *ART* antiretroviral therapy, *ATT* anti-tuberculous therapy, *IQR* interquartile range, *LAM* lipoarabinomannan, *SD* standard deviation, *TB* tuberculosis

^1^Empiric ATT

^2^*P* values compare all four subgroups with the exception of AFB smear positive and *M. tuberculosis* culture positive where active TB and subclinical TB only are compared

^3^ART was offered to all participants according to contemporaneous South African Department of Health and WHO guidelines and initiated at CD4 ≤350 cells/mm^3^ for groups with TB disease (included here as active and subclinical TB as well no microbiologic TB/ATT) and CD4 ≤200 cells/mm^3^ for no microbiologic TB/no ATT. CD4 data was available for 567 of 654 participants

Ninety six (15%) participants were diagnosed with active TB disease, 28 (4%) with subclinical TB disease, and 530 did not have microbiologic evidence of TB. Of those without proven disease, 40 (6%) were empirically treated with multi-drug ATT. There were no significant differences in mean age, education, marital status, baseline tobacco consumption, or prior TB treatment between the four subgroups. The active TB group had the highest proportion of men (68%, *P 0.02*).

Median CD4 count differed across groups: 68 (IQR 39–161) cells/mm^3^ in active TB, 136 (IQR 72–312) cells/mm^3^ in subclinical TB, 90 (IQR 40–180) cells/mm^3^ in no microbiologic TB/ATT and 249 (125–394) cells/mm^3^ in no microbiologic TB/no ATT, (*P < 0.001*). In addition to having a lower median CD4, the no microbiologic TB/ATT group had a higher burden of TB-related symptoms than the no microbiologic TB/no ATT group. According to baseline CD4 count and TB diagnosis, persons eligible for ART included 79 (89%) with active TB, 22 (81%) with subclinical TB, 30 (77%) with no microbiologic TB/ATT, and 157 (38%) with no microbiologic TB/no ATT (*P < 0.001)*. Twenty-nine percent of individuals in the subclinical group were AFB smear positive compared to 14% in the active TB group (*P 0.08*). The proportion positive for urine LAM also differed across groups: 37% in active TB, 25% in subclinical TB, 23% in no microbiologic TB/ATT, and 10% in no microbiologic TB/no ATT (*P < 0.001*).

**Fig. 1:**
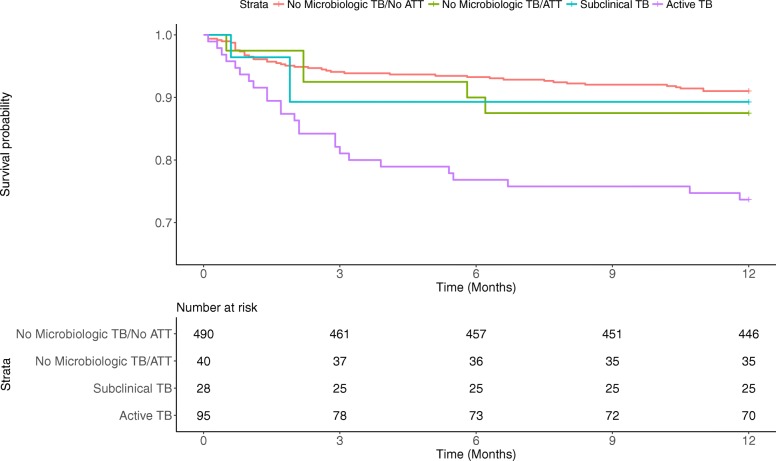
Kaplan Meier survival curves of adults with HIV stratified by tuberculosis status

Vital status at 12 months was ascertained in all participants and death was documented in 25 (26%) of active TB, 3 (11%) of subclinical TB, 5 (13%) of no microbiologic TB/ATT, and 44 (9%) of no microbiologic TB/no ATT individuals (Table 2, Fig. 1). In univariate analysis, male sex, older age, and lower baseline CD4 count were associated with a higher hazard of death. Subclinical TB disease was associated with a similar hazard of death at 12 months compared to the reference no microbiologic TB/no ATT group (hazard ratio [HR] 1.22, 95% CI 0.38–3.93) while active TB disease was associated with a higher hazard of death compared to the reference group (HR 3.23, 95% CI 1.97–5.27). After adjusting for age, sex, and baseline CD4, there remained no evidence of a significant association between hazard of death comparing subclinical TB to reference (adjusted hazard ratio [aHR] 0.84, 95% CI 0.26–2.73), and the increased hazard of death comparing active TB to reference was attenuated and no longer significant (aHR 1.55, 95% CI 0.90–2.66). There was a nearly two-fold non-significant difference in 12-month survival comparing active to subclinical TB (aHR 1.84, 95% CI 0.55–6.17).

**Table 2:** Cox proportional hazards stratified by tuberculosis status

	Active TB *N=96*	Subclinical TB *N=28*	No Microbiologic TB/ATT^1^ *N=40*	No Microbiologic TB/ No ATT *N=490*
Death at 12 months	25 (26%)	3 (11%)	5 (13%)	44 (9%)
Unadjusted HR (95% CI)	3.23 (1.97, 5.27)	1.22 (0.38, 3.93)	1.40 (0.56, 3.54)	Reference
*P* value^2^	*<0.001*	*0.74*	*0.47*	
Adjusted HR (95% CI)^3^	1.55 (0.90, 2.66)	0.84 (0.26, 2.73)	0.74 (0.29, 1.91)	--
*P* value^2^	*0.11*	*0.77*	*0.53*	
Unadjusted HR (95% CI)	2.60 (0.78, 8.63)	Reference		
*P* value^4^	*0.12*			
Adjusted HR (95% CI)^3^	1.84 (0.55, 6.17)	--		
*P* value^4^	*0.32*			

Abbreviations: *ATT* anti-tuberculous therapy, *CI* confidence interval, *HR* hazard ratio, *TB* tuberculosis

^1^Empiric ATT treatment

^2^*P* values correspond to hazard ratios comparing survival to no microbiologic TB not treated reference group

^3^Adjusted for age, sex, CD4

^4^*P* values correspond to hazard ratios comparing survival to subclinical TB group

Mortality outcomes were also examined in comparison to a reference group comprised of 442 individuals without microbiologic TB who also tested LAM negative and did not receive empiric ATT. The unadjusted hazard of death at 12 months comparing the subclinical TB group to this LAM negative reference was similar to earlier findings presented in Table 2 (HR 1.49, 95% CI 0.46–4.85) as was the hazard of death comparing the active TB group to the LAM negative reference (HR 3.93, 95% CI 2.34–6.61). After adjusting for age, sex, and baseline CD4, there remained no evidence of a significant association between hazard of death comparing subclinical TB to LAM negative reference (aHR 1.02, 95% CI 0.31–3.38). However, while the increased hazard of death comparing active TB to LAM negative reference was again attenuated, it remained significant (aHR 1.93, 95% CI 1.08–3.47). Finally, adjusted models stratified by LAM test result did not show a significant association between hazard of death comparing either subclinical or active TB groups to LAM negative reference.

Information on whether ATT was initiated was available for half of all TB cases: 15 of 28 individuals in the subclinical TB group and 48 of 96 individuals in the active TB group were known to start multi-drug ATT, while treatment initiation in the remaining individuals was not documented. Survival outcomes in these individuals were also examined in comparison to the no microbiologic TB/no ATT reference group. Treated subclinical TB disease was associated with a similar hazard of death at 12 months compared to the reference group (HR 0.73, 95% CI 0.10–5.33) while treated active TB disease was associated with a higher hazard of death compared to the reference group (HR 2.12, 95% CI 1.03–4.34). After adjusting for age, sex, and baseline CD4, there remained no evidence of a significant association between hazard of death comparing treated subclinical TB to reference (aHR 0.51, 95% CI 0.07–3.74), and there was no longer a significant difference in hazard of death comparing treated active TB to reference (aHR 0.88, 95% CI 0.41–1.88). There was a two-fold non-significant difference in 12-month survival comparing active to subclinical TB (aHR 2.01, 95% CI 0.24–16.80).

## Discussion

The prevalence of subclinical TB in our population of untreated PLHIV in South Africa was high, accounting for 23% of all TB cases and 4% of the entire screened population. Subclinical TB cases presented with an intermediate degree of immunosuppression as reflected by a median CD4 count between those with active disease and no microbiologic TB/no ATT. We did not find evidence of reduced survival in adjusted models comparing subclinical or active TB to no microbiologic TB/no ATT individuals. This is one of the largest series of subclinical TB to date.

The high prevalence of subclinical TB among all screened participants in our study is consistent with other untreated HIV-infected populations. Subclinical disease has been reported to account for 6–52% of TB cases diagnosed by sputum culture [9, 10, 18–29]. Despite this, it remains an underrecognized entity among PLHIV.

The degree of AFB smear positivity may have important implications for potential transmission [30], particularly as PLHIV with subclinical TB disease can progress to being symptomatic within several days to months [10, 24, 25]. Though we did not find a significant difference in the proportion of smear positive cases between subclinical and active TB groups, adequate power to detect such a difference was limited by sample size. Relative patterns in the literature have been variable, with one group describing greater smear positivity in subclinical disease [9] and others reporting greater bacillary burden and shorter time to culture positivity in active TB disease [10, 31]. The overall contribution of subclinical disease to TB transmission is not well understood.

The observed pattern of intermediate immunosuppression in subclinical TB is not consistently seen in all pre-ART cohorts. Some studies have reported similar CD4 values between groups [9] while others have described a notable difference [10].

With regard to survival outcomes, the study was not powered to detect a difference in adjusted survival hazards across groups. Baseline CD4 was a significant confounder; lower counts were strongly associated with increased hazard of death. Importantly, we did not fully measure ATT, ART, or treatment adherence, all of which impact survival. To address this issue, we performed subanalysis adjusting for ART eligibility according to TB diagnosis and baseline CD4 count. Persons with active TB, subclinical TB, or who were empirically treated for TB were considered eligible if their CD4 count was ≤350, while persons without microbiologic evidence of TB who did not receive ATT were considered eligible if their CD4 count was ≤200. Mortality outcomes did not differ significantly from main analysis. Furthermore, in subanalysis of subclinical and active TB groups documented to start ATT, mortality outcomes did not differ significantly from main analysis, though the adjusted hazard of death comparing active TB to reference was further attenuated albeit non-significant. A number of individuals with subclinical TB were started on ATT which was likely influenced by knowledge of culture results; in routine clinical practice where cultures are not obtained in the absence of symptoms, survival among subclinical TB patients may differ.

Diagnostic limitations of our study include collection and testing of only a single sputum sample per patient and lack of radiologic data. In addition, a number of adults without evidence of TB by sputum testing were urine LAM test positive; this finding could indicate extrapulmonary or undiagnosed pulmonary TB. To address potential misclassification, we performed subanalysis using a LAM negative no microbiologic TB reference group. Adjusted mortality outcomes were similar, though the increased hazard of death seen in the active TB group compared with reference remained significant.

Finally, since this study was conducted, HIV treatment guidelines have shifted to reflect the benefit of early ART initiation with regard to mortality, morbidity, and HIV transmission [32–35]. With wider ART coverage, TB-related mortality has been reduced [36], though the impact on the epidemiology and natural history of subclinical TB remains unknown.

## Conclusions

We report a high prevalence of subclinical TB in our cohort of HIV-infected adults in South Africa. These individuals were characterized by an intermediate degree of immunosuppression, and although no differences in mortality were observed, multi-drug ATT was commonly started in those with subclinical TB. Given the potential impact on morbidity and mortality, particularly among PLHIV, early identification of subclinical TB is critical.

## Abbreviations

AFB: Acid-fast bacilli
aHR: adjusted hazard ratio
ART: antiretroviral therapy
ATT: anti-tuberculous therapy
CI: confidence interval
HR: hazard ratio
IQR: interquartile range
LAM: lipoarabinomannan
MGIT: mycobacterial growth indicator tubes
PLHIV: persons living with HIV
TB: tuberculosis
